# Rarefaction is currently the best approach to control for uneven sequencing effort in amplicon sequence analyses

**DOI:** 10.1128/msphere.00354-23

**Published:** 2024-01-22

**Authors:** Patrick D. Schloss

**Affiliations:** 1 Department of Microbiology & Immunology, University of Michigan, Ann Arbor, Michigan, USA; University of Wisconsin-Madison, Madison, Wisconsin, USA

**Keywords:** microbial ecology, bioinformatics, amplicon sequencing, 16S rRNA gene sequencing, microbiome, data science

## Abstract

Considering it is common to find as much as 100-fold variation in the number of 16S rRNA gene sequences across samples in a study, researchers need to control for the effect of uneven sequencing effort. How to do this has become a contentious question. Some have argued that rarefying or rarefaction is “inadmissible” because it omits valid data. A number of alternative approaches have been developed to normalize and rescale the data that purport to be invariant to the number of observations. I generated community distributions based on 12 published data sets where I was able to assess the ability of multiple methods to control for uneven sequencing effort. Rarefaction was the only method that could control for variation in uneven sequencing effort when measuring commonly used alpha and beta diversity metrics. Next, I compared the false detection rate and power to detect true differences between simulated communities with a known effect size using various alpha and beta diversity metrics. Although all methods of controlling for uneven sequencing effort had an acceptable false detection rate when samples were randomly assigned to two treatment groups, rarefaction was consistently able to control for differences in sequencing effort when sequencing depth was confounded with treatment group. Finally, the statistical power to detect differences in alpha and beta diversity metrics was consistently the highest when using rarefaction. These simulations underscore the importance of using rarefaction to normalize the number of sequences across samples in amplicon sequencing analyses.

**IMPORTANCE:**

Sequencing 16S rRNA gene fragments has become a fundamental tool for understanding the diversity of microbial communities and the factors that affect their diversity. Due to technical challenges, it is common to observe wide variation in the number of sequences that are collected from different samples within the same study. However, the diversity metrics used by microbial ecologists are sensitive to differences in sequencing effort. Therefore, tools are needed to control for the uneven levels of sequencing. This simulation-based analysis shows that despite a longstanding controversy, rarefaction is the most robust approach to control for uneven sequencing effort. The controversy started because of confusion over the definition of rarefaction and violation of assumptions that are made by methods that have been borrowed from other fields. Microbial ecologists should use rarefaction.

## INTRODUCTION

The ability to generate millions of 16S rRNA gene amplicon and metagenomic sequence reads has allowed researchers to multiplex multiple samples on the same sequencing run by pooling separate PCRs that can be deconvoluted later based on index (aka barcode) sequences that are embedded into the sequence of the PCR primers (1–3). Unfortunately, it is common to observe variation in the number of sequence reads from each sample vary by as much as 100-fold (e.g., see Fig. S1). This occurs because pooling of DNA from multiple PCRs is fraught with numerous opportunities for random sampling errors to compound leading to a skewed distribution. Aside from developing better methods of pooling DNA, the question of how to control for uneven sequencing effort in microbial ecology studies has become controversial.

The practice of rarefaction has been commonly used in ecology for more than 50 years as a tool to control for uneven sequencing effort across experimental replicates (4, 5). Microbial ecologists have used it to compare 16S rRNA gene sequence data for the past 25 years (6–8). With rarefaction, the investigator selects a desired threshold of sequencing effort and removes any samples below that threshold. They then randomly select that many sequences, without replacement from each sample. Based on the observed sequence counts, the researcher can then calculate alpha diversity metrics including richness and diversity indices or beta diversity metrics such as a Jaccard or Bray-Curtis dissimilarity index. I refer to this single sampling as a subsample; this method is implemented as the sub.sample function in mothur (9) and the rrarefy function in the vegan R package (10). Rarefaction repeats the subsampling a large number of times (e.g., 100 or 1,000 times) and calculates the mean of the alpha or beta diversity metric over those subsamplings; rarefaction is implemented in mothur using the summary.single and dist.shared functions (9) and with the vegan R package using the rarefy or avgdist functions (10). Rarefaction effectively tells a researcher what an alpha or beta diversity metric would have been for a collection of samples if they were all sequenced to the same depth. Although a closed form equation exists to calculate the expected richness (5), it is computationally easier to empirically calculate richness and other alpha and beta diversity metrics by rarefaction.

In 2014, McMurdie and Holmes (11) announced that “rarefying” of microbial community data was “statistically inadmissible” because it omits valid data. In their simulations, they observed that rarefying reduced the statistical power to correctly cluster samples into the same treatment groups based on beta diversity metrics. I have recently shown that their analysis had a number of important problems (12). First, although the detail was lost on many subsequent researchers, they did not describe rarefaction. Instead, they described rarefying as a single subsampling of an operational taxonomic unit (OTU) abundance table whereas rarefaction repeats the subsampling step many times. Furthermore, their simulations penalized the rarefied data by removing 15% of the samples but did not remove those samples when considering their other methods. They also evaluated the accuracy of clustering samples using a clustering algorithm that performed worse for rarefied data. According to my reanalysis of rarefaction using their original simulation framework, but using all of the samples, rarefaction outperformed the other normalization methods they described for both alpha and beta diversity metrics (12). Others have also critiqued the original work (13).

Since McMurdie and Holmes published their simulations, others have developed alternative approaches to control for uneven sequencing effort in amplicon sequencing studies. For alpha diversity metrics, Willis used overly simplistic data sets to demonstrate that one could estimate the richness for each sample in a data set and use those values for statistical comparisons (14). Non-parametric estimators of the minimum richness in a community and diversity (15–17) and parametric estimators of richness (18) have been used in microbial ecology studies. One non-parametric approach, iNEXT, combines rarefaction on samples more than a threshold number of sequences and extrapolation on samples below the threshold (17). This method has become popular in traditional ecology studies, but has not been widely used among microbial ecologists. For beta diversity metrics, at least four approaches have been pursued as alternatives to rarefaction. First, one could use relative abundance values where the observed number of sequences in an OTU is divided by the total number of sequences in the sample (19). Second, normalization strategies have been developed where the relative abundance is multiplied by the size of the minimum desired sequencing effort and fractional values are reapportioned to the OTUs to obtain integer values (20, 21). Third, a variety of center log-ratio methods have been developed where the compositional nature of the OTU counts is removed and used to calculated Euclidean distances (aka Aitchison distances) (19, 21–24). This strategy is purported to control for uneven sequencing effort (23, 25); however, some have noticed that this feature breaks down under certain conditions (26). Finally, variance stabilization transformations have been recommended to generate values that can be used to calculate Euclidean distances (11).

The ongoing controversy over the use of rarefaction and the recent development of alternative strategies to control for uneven sequencing effort caused me to question how these methods compared to each other using a simulation framework that overcame the issues with the McMurdie and Holmes study (11). My analysis included 16S rRNA gene sequence data from 12 diverse environments (Table 1; Fig. S1). The sequences were assigned to OTUs using a standard pipeline and their frequencies and the number of sequences found in each sample were used to generate simulated communities and treatment effects. For each data set and simulation, 100 replicate data sets were generated and used as input to each of the strategies for controlling for uneven sequencing effort. My overall conclusion was that rarefaction outperformed the alternative strategies.

**TABLE 1:** Summary of studies used in the analysis^*a*^

Data set (reference)	Samples	Total sequences	Median sample size	Mean sample size	Range of sample sizes	BioProject accession no.
Bioethanol (27)	95	3,970,972	16,014	41,799	3,690–356,027	PRJNA276052
Human (28)	490	20,828,275	32,452	42,506	10,439–422,904	PRJNA290926
Lake (29)	52	3,145,486	69,205	60,490	15,135–110,993	PRJNA255432
Marine (30)	7	1,484,068	213,091	212,009	132,895–256,758	PRJNA307596
Mice (3)	348	2,785,641	6,426	8,004	1,804–30,311	PRJNA531928
Peromyscus (31)	111	1,545,288	12,393	13,921	4,454–33,502	PRJNA254334
Rainforest (32)	69	936,666	11,464	13,574	4,880–37,403	PRJEB21491
Rice (33)	490	22,623,937	43,399	46,171	2,777–192,200	PRJNA255789
Seagrass (34)	286	4,135,440	13,538	14,459	1,830–45,076	PRJNA350672
Sediment (35)	58	1,151,389	17,606	19,851	7,686–67,763	PRJNA362622
Soil (36)	18	932,563	50,487	51,809	46,622–58,935	PRJEB10725
Stream (37)	201	21,017,610	90,621	104,565	8,931–394,419	PRJNA323602

^*a*^ For all studies, when rarefaction was used, the number of sequences used from each data set was the size of the smallest sample. A graphical representation of the distribution of sample sizes for each data set and the samples that were removed from each data set are provided in Fig. S1. This table is similar to Table 1 from reference 38.

## RESULTS

### Without rarefaction, metrics of alpha diversity are sensitive to sequencing effort

**Fig 1:**
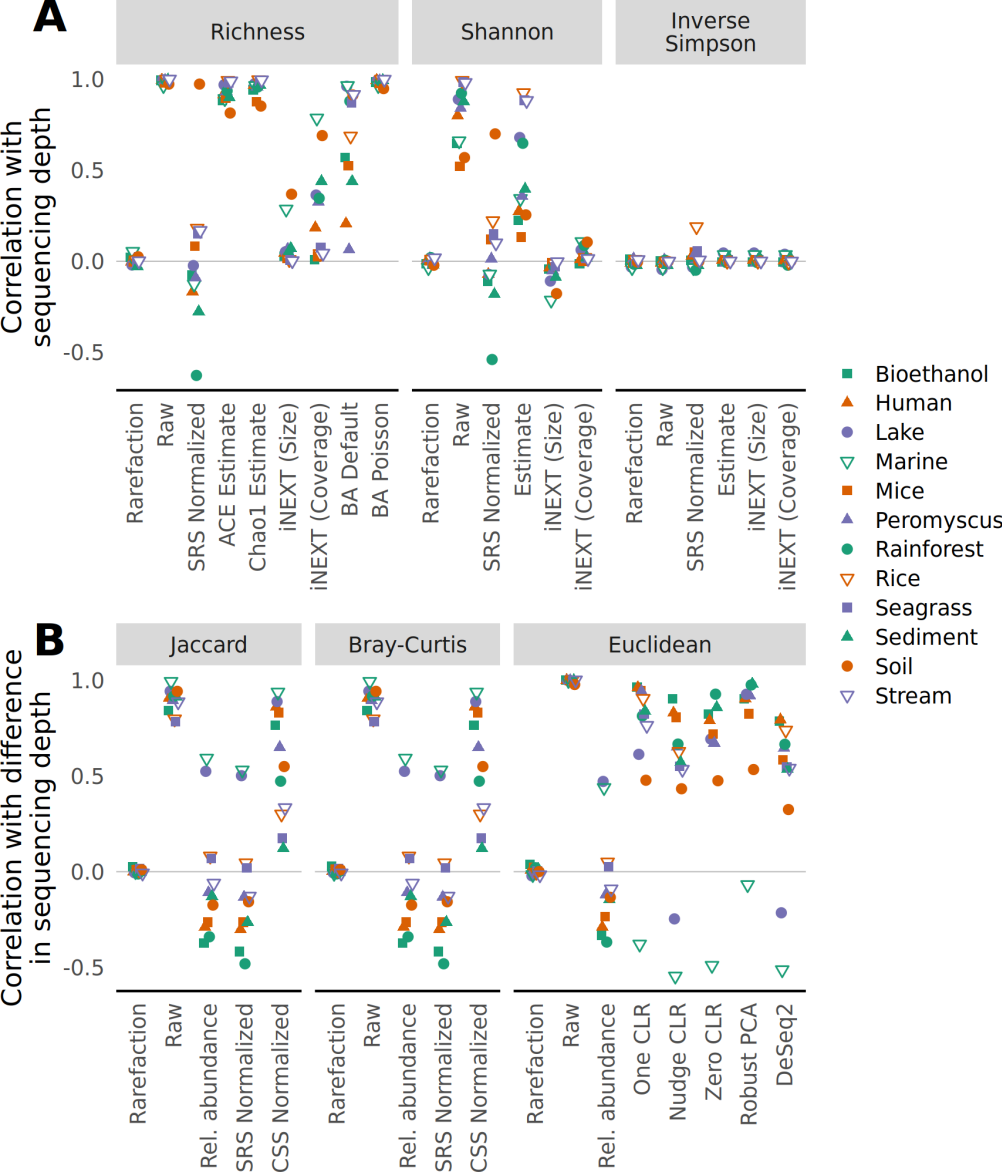
Rarefaction eliminates the correlation between sequencing depth and alpha diversity (**A**) and between differences in sequencing depth and beta (**B**) diversity metrics when using null community models. Examples of the relationship between different metrics and methods for controlling for uneven sequencing effort are provided in Fig. S2 and S3 for alpha and beta diversity metrics, respectively. Each point represents the mean of 100 random null community models; the standard deviation was smaller than the size of the plotting symbol.

To test the sensitivity of various approaches of measuring alpha diversity to sequencing effort, I generated null models using OTU counts and sequencing depths from 12 studies. Under a null model, each community from the same data set would be expected to have the same alpha diversity regardless of the sequencing effort. I measured the richness of the communities in each data set without any correction, using scaled ranked subsampling (SRS) normalized OTU counts, with estimates based on non-parametric and parametric approaches, and using rarefaction (e.g., Fig. S2). For each data set, all of the approaches, except for rarefaction and possibly the size-based iNEXT estimates, showed a strong correlation between richness and the number of sequences in the sample (Fig. 1A). The size-based iNEXT estimates were correlated with sampling effort for the Marine and Soil data sets, which had fewer samples and more uniform sequencing depth distributions than the other data sets. Next, I assessed diversity using the Shannon diversity index and the inverse Simpson diversity index without any correction, using normalized OTU counts, non-parametric estimation, and rarefaction. The correlation between sequencing depth and the Shannon diversity metric was not as strong as it was for richness. The inverse Simpson diversity values was not sensitive to sampling effort (Fig. 1A). The alpha-diversity metrics calculated with rarefaction consistently demonstrated a lack of sensitivity to sequencing depth.

### Without rarefaction, metrics of beta diversity are sensitive to sequencing effort

To test the sensitivity of various approaches of measuring beta diversity to sequencing effort, I used the same null models used for studying the sensitivity of alpha diversity. Under a null model, the ecological distance between any pair of samples would be the same regardless of the difference in the number of sequences observed in each sample (e.g., Fig. S3). First, I calculated the Jaccard distance coefficient between all pairs of communities within a data set. The Jaccard distance coefficient is the fraction of OTUs that are unique to either community and does not account for the abundance of the OTUs. Jaccard distances were calculated using the uncorrected OTU counts, with rarefaction, relative abundances, and following normalization using cumulative sum scaling (CSS) and SRS. Only the distances calculated with rarefaction showed a lack of sensitivity to sequencing effort (Fig. 1B). Second, I analyzed the sensitivity of the Bray-Curtis distance coefficient, which is a popular metric that incorporates the abundance of each OTU. Similar to what I observed with the Jaccard coefficient, only the data normalized with rarefaction showed a lack of sensitivity to sequencing effort (Fig. 1B). Third, I calculated the Euclidean distance on raw OTU counts where the central log-ratio (CLR) was calculated (i.e., Aitchison distances) by adding a pseudo-count of one to all OTU counts prior to calculating the CLR (One CLR), adding a pseudo-count of one divided by the total number of sequences obtained for the community (Nudge CLR), imputing the value of zero counts (Zero CLR), and using CLR on non-zero counts followed by matrix completion on zero counts (robust principle components analysis [PCA]). The Aitchison distances were all strongly sensitive to sequencing effort (Fig. 1B). Finally, I used the variance-stabilization transformation (VST) from DESeq2 prior to calculating Euclidean distances. Again, there was a strong sensitivity to sequencing effort (Fig. 1B). Although use of Euclidean distances is not advised with raw or rarefaction-normalized count data in ecology (39), Euclidean distances calculated with rarefaction were not sensitive to sequencing effort. Across each of the beta diversity metrics and approaches used to account for uneven sequencing effort and sparsity, rarefaction was the least sensitive approach to differences in sequencing effort.

### Rarefaction limits the detection of false positives when sequencing effort and treatment group are confounded

**Fig 2:**
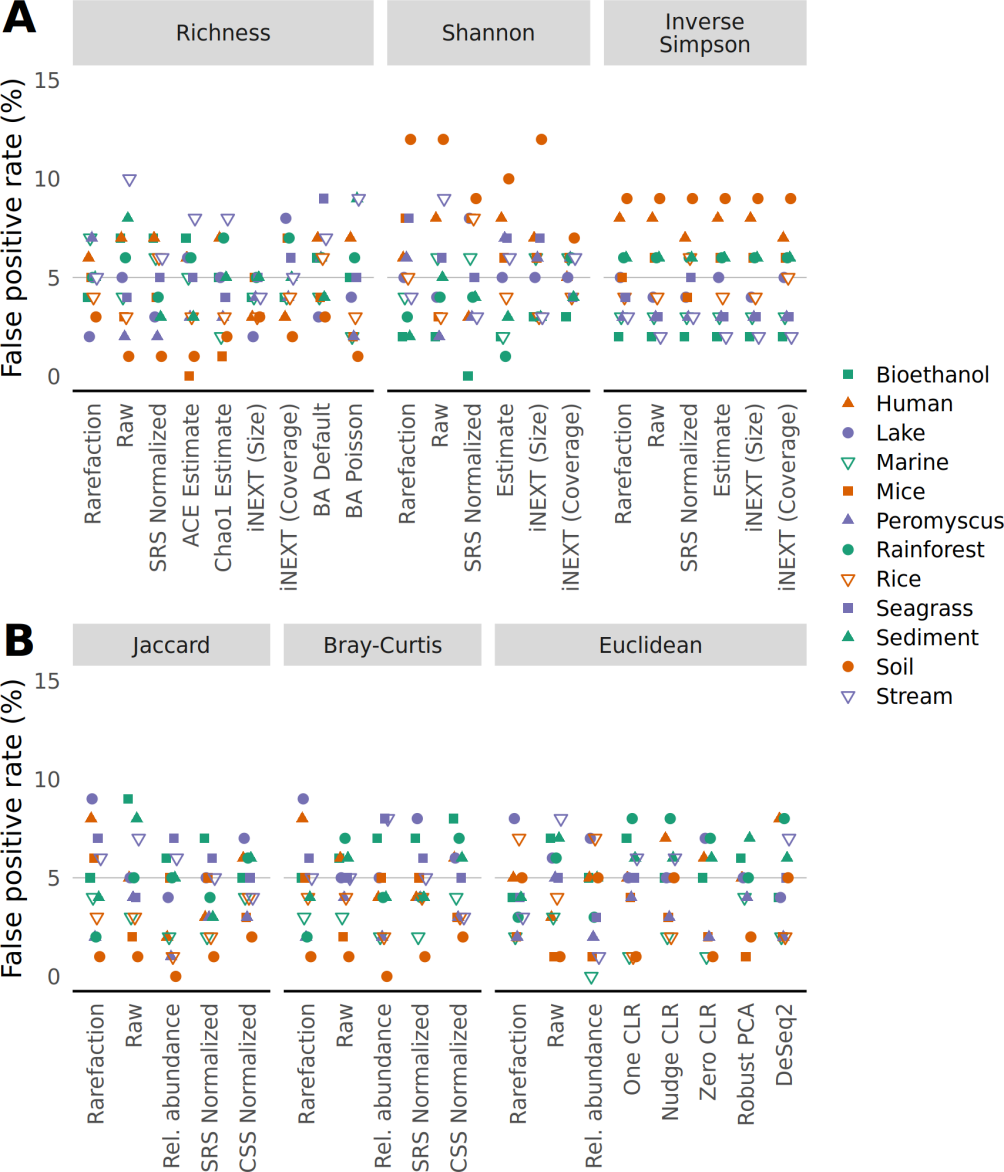
The risk of falsely detecting a difference between treatment groups drawn from a null model does not meaningfully vary from 5%, regardless of approach for controlling for uneven sequencing depth. Samples were randomly assigned to different treatment groups. To calculate the false detection rate, data sets were regenerated 100 times and differences in alpha diversity were tested using a Wilcoxon test (**A**) and differences in beta diversity were tested using PERMANOVA (**B**) at a 5% threshold. The false positive rate was the number of times a data set yielded a significant result.

**Fig 3:**
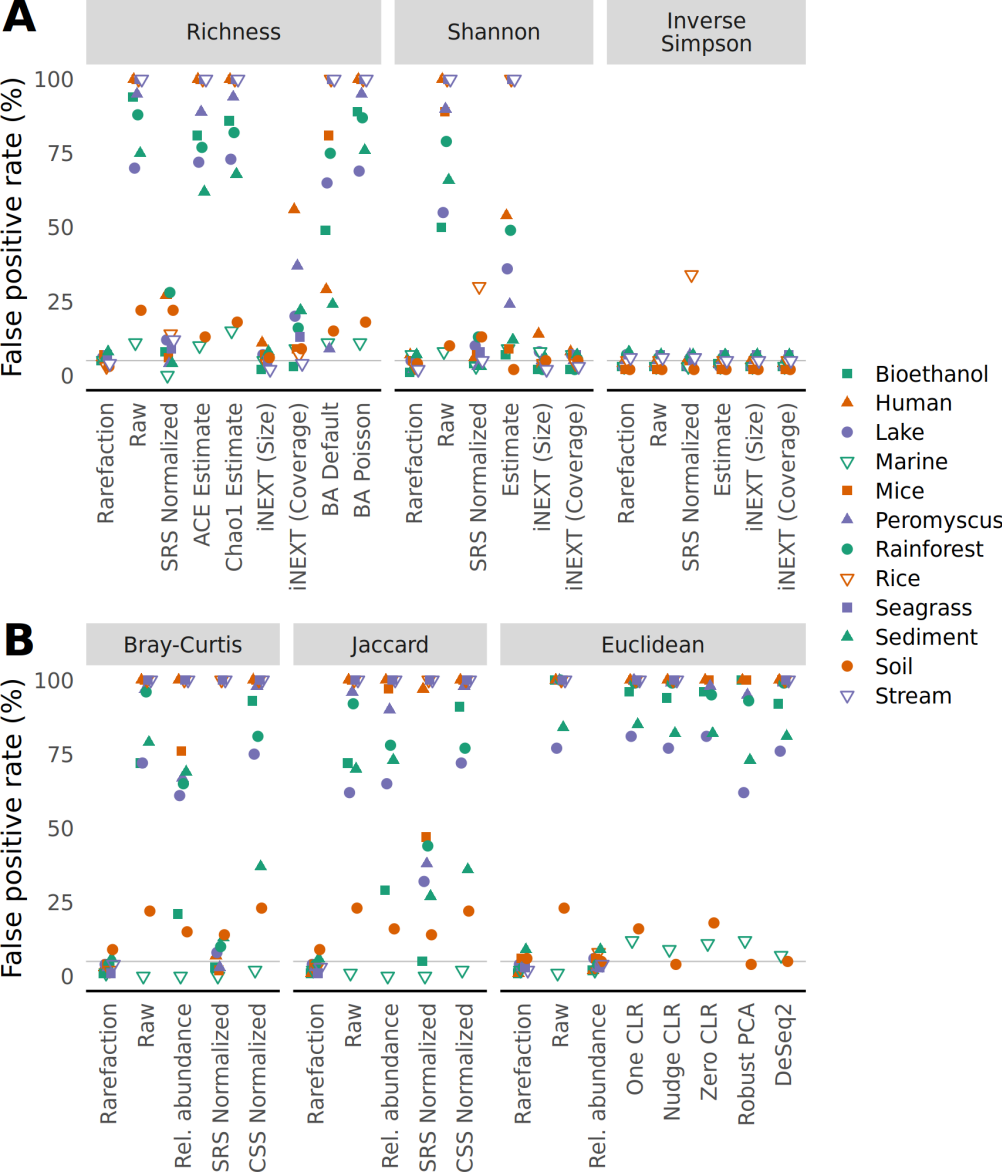
The risk of falsely detecting a difference between treatment groups drawn from a null model did not meaningfully vary from 5% when data are normalized by rarefaction when sequencing depth was confounded with treatment group. Samples were assigned to different treatment groups so that the smallest 5% of samples were assigned to one treatment group and the largest 5% to another; the other 90% of samples were randomly assigned to both treatment groups. To calculate the false detection rate, data sets were regenerated 100 times and differences in alpha diversity were tested using a Wilcoxon test (**A**) and differences in beta diversity were tested using PERMANOVA (**B**) at a 5% threshold. The false positive rate was the number of times a data set yielded a significant result.

Next, I investigated the impact of the various strategies and metrics on falsely detecting a significant difference using the the same communities generated from the null model in the analysis of alpha and beta diversity metrics. To test for differences in alpha and beta diversity, I used the non-parametric Wilcoxon test and non-parametric permutation-based multivariate analysis of variance (PERMANOVA). First, I employed an unbiased null treatment model to measure the false detection rate, which should not have meaningfully differed from 5%. Indeed, for each data set and alpha and beta diversity metric and strategy for accounting for uneven sequencing, approximately 5% of the tests yielded a significant result (Fig. 2). Second, I employed a biased null treatment model where the treatment group was confounded with the number of sequences in each sample such that the 50% of samples with the least sequencing effort were in one treatment group and the others were in another treatment group (Fig. S4). Under this model, only the data normalized with rarefaction consistently resulted in a 5% false positive rate for alpha and beta diversity metrics. I then used a less severe bias where the smallest 5% of samples were assigned to one treatment group and the largest 5% were assigned to another; the other samples were randomly assigned to both treatment groups. Although the sensitivity of the false positive rate to the level of confounding varied across methods of quantifying alpha and beta diversity, rarefaction and the iNEXT size-based estimates for alpha diversity were the only methods to consistently control the false positive rate (Fig. 3). These results aligned with the observed sensitivity of alpha and beta diversity metrics to sequencing effort and underscore the value of rarefaction.

### Rarefaction preserves the statistical power to detect differences between treatment groups

**Fig 4:**
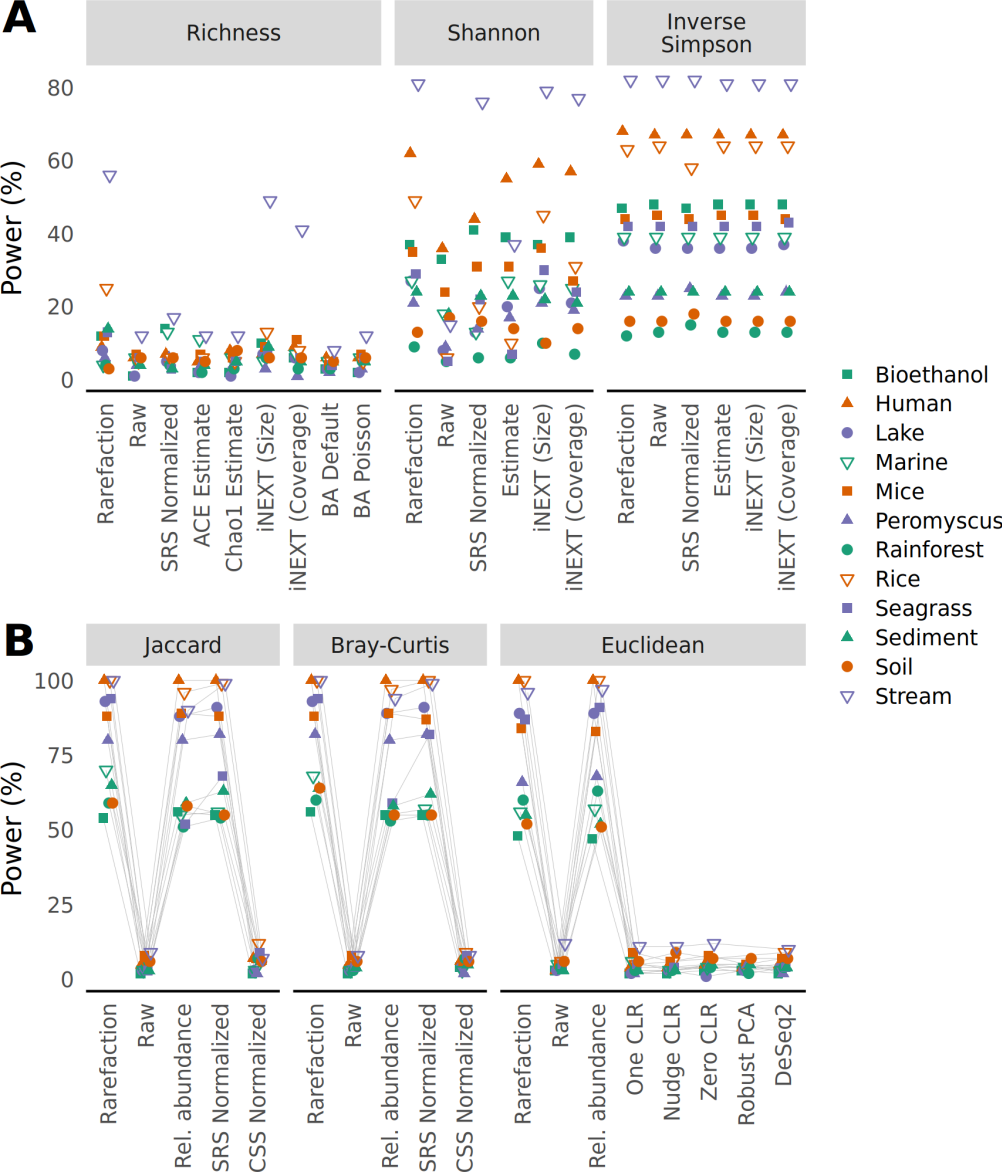
The ability to detect true differences in treatment groups for alpha (**A**) and beta (**B**) diversity metrics is greatest when communities differing in the relative abundance of their OTUs are normalized by rarefaction. For each data set, samples were randomly assigned to one of two community distributions where the abundance of OTUs differed. To calculate the power for each data sets, data sets were regenerated 100 times and differences in alpha diversity were tested using a Wilcoxon test (**A**) and differences in beta diversity were tested using PERMANOVA (**B**) at a 5% threshold. The power was the number of times a data set yielded a significant result.

**Fig 5:**
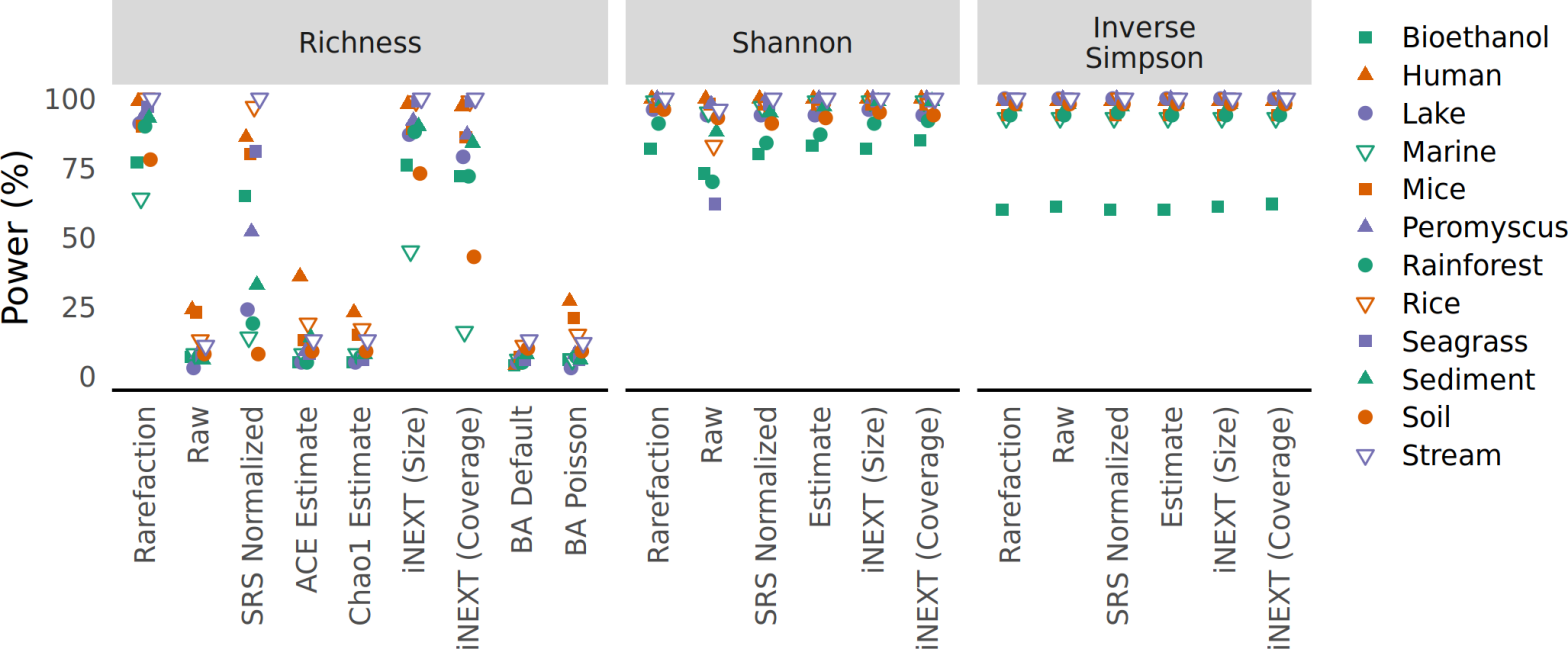
The ability to detect true differences in treatment groups for alpha diversity metrics is greatest when sequencing depths from communities differing in richness are normalized by rarefaction. For each data set, samples were randomly assigned to one of two community distributions where one distribution contained a subset of OTUs found in the other. To calculate the power for each data set, data sets were regenerated 100 times and differences in alpha diversity were tested using a Wilcoxon test (**A**) and differences in beta diversity were tested using PERMANOVA (**B**) at a 5% threshold. The power was the number of times a data set yielded a significant result.

To assess the impact of different approaches to control for uneven sequencing effort, I performed two additional simulations. In the first simulation, I implemented a skewed abundance distribution model to create two treatment groups for each data set that were each populated with half of the samples each with the same number of sequences as the samples had in the observed data. The two treatment groups varied in their structure such that one had the same abundances as the null distribution above and the other had 10% of its OTUs randomly selected to increase their counts by 5%. The power to detect differences in richness between the two simulated treatment groups by all approaches was low (Fig. 4A). This was likely because the approach for generating the perturbed community did not necessarily change the number of OTUs in each treatment group; the differences primarily affected the distribution of the sequences across OTUs. Regardless, the simulations testing differences in richness using the Rice and Stream data sets had the greatest power when the richness data were calculated with rarefaction. To explore this further, a richness-adjusted community model was created by randomly selecting and removing 3% of the OTUs from a null model. As suggested by the first simulation, the richness data calculated with rarefaction consistently had the highest statistical power among the approaches used to measure richness followed by the iNEXT size-based estimates (Fig. 5). Returning to simulations where treatment groups varied in their structure, the power to detect differences in Shannon diversity was the lowest when using raw relative abundances than when using the other methods (Fig. 4A and 5). When testing for differences in the inverse Simpson diversity index, the the difference between rarefaction and the other methods was negligible (Fig. 4A and 5). For tests of beta diversity, I found that rarefaction was the most reliable approach to maintain statistical power to detect differences between two communities (Fig. 4B). Among the tests using the Jaccard and Bray-Curtis metrics, raw count data and CSS normalized data had little power relative to using rarefaction, relative abundance, and SRS to normalize the uneven sequencing depths. The differences in power between counts normalized with rarefaction, relative abundance, and SRS data were small, but if there were differences, the power obtained using rarefaction was greater than the other methods. Among the tests using Euclidean distances, using raw counts and CLR and DESeq2 transformed data had little power relative to the distances calculated using rarefaction and relative abundances. This power-based analysis of the simulated communities using different methods of handling uneven sample sizes demonstrated the value of rarefaction for preserving the statistical power to detect differences between treatment groups for measures of alpha and beta diversity.

### Increased rarefaction depth reduces intra-sample variation in alpha and beta diversity

**Fig 6:**
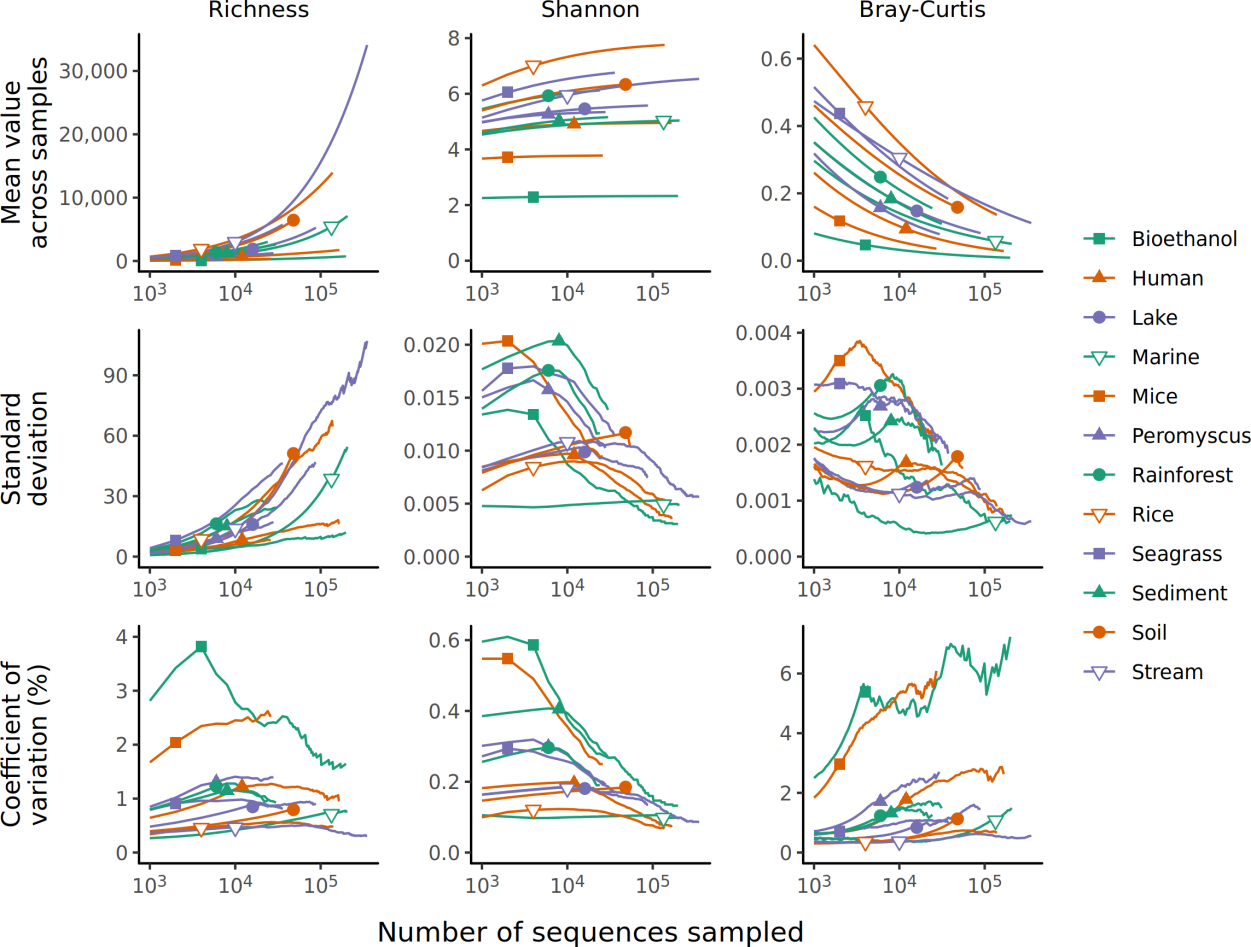
The mean and coefficient of variation for richness, Shannon diversity, and Bray-Curtis dissimilarity values calculated by rarefaction vary with sequencing depth. For each data set, a null community distribution was created and samples were created to have the same sequencing depth as they did originally. The placement of the plotting symbol indicates the size of the smallest sample. Results are only shown for sequencing depths where a data set had five or more samples.

One concern with using rarefaction is the perceived loss of sequencing information when a large fraction of data appears to be removed when the community with the greatest sequencing depth is sampled to the size of the community with the least (e.g., the smallest sample in the Bioethanol data set had 1.04% of sequences that were in the largest sample). To assess the sensitivity of alpha and beta diversity metrics to rarefaction depth, I again used the data set generated using the null models, but used rarefaction with each community to varying sequencing depths (Fig. 6). The richness values increased with sequencing effort as rare OTUs would continue to be detected. In contrast, the Shannon diversity and Bray-Curtis values plateaued with increased sequencing effort. This result was expected since increased sequencing would lead to increased precision in the measured relative abundance of the OTUs. Next, I measured the coefficient of variation (i.e., the mean divided by the standard deviation) between samples for richness, Shannon diversity, and Bray-Curtis distances. Although the mean richness appeared to increase unbounded with sequencing effort, the coefficient of variation for each data set was relatively stable. In general, the coefficient of variation increased slightly with sequencing depth only to decline once smaller samples were removed from the analysis at higher sequencing depths. Interestingly, the coefficient of variation between Shannon diversity values decreased toward zero with increased sequencing effort, and the coefficient of variation between Bray-Curtis distances tended to increase slightly for samples where the standard deviation did not decrease as quickly as the mean distance between samples. Regardless, the coefficients of variation were relatively small. This analysis indicates that there are benefits to increased sequencing depths.

### Most studies have a high level of sequencing coverage

**Fig 7:**
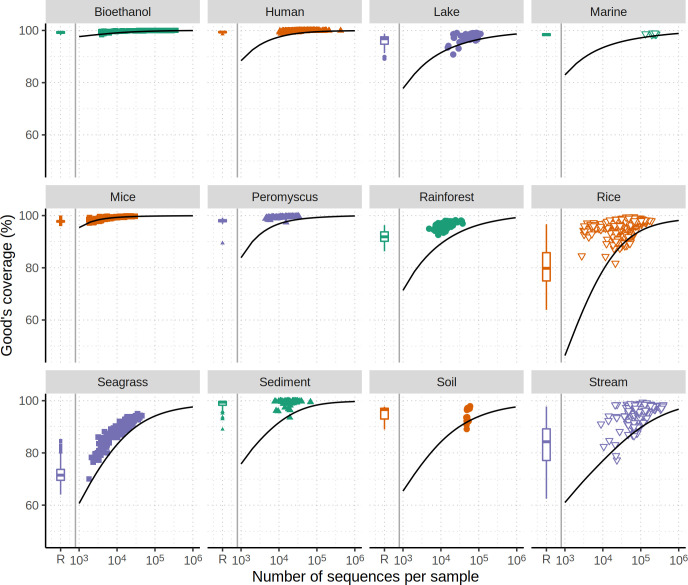
Most data sets are sequenced to a level that provides a high level of coverage. Each plotting symbol represents the observed Good’s coverage for a different sample in each data set. The box and whisker plot indicates the range of coverage values when the observed community data were normalized by rarefaction to the size of the least sequenced sample. The smoothed line indicates the simulated coverage for varying levels of sequencing effort when a null community is generated from the observed data.

To explore the concern over loss of sequencing depth further, I calculated the Good’s coverage for the observed data. The median coverage for each data set ranged between 89.4% and 99.8% for the Seagrass and Human data sets, respectively (Fig. 7). When I used a rarefaction threshold with each data set at the size of the smallest community in the data set, with the exception of the Seagrass, Rice, and Stream data sets, the median coverage with rarefaction was still greater than 90% (see the box plots in Fig. 7). These results suggest that most studies had a level of sequencing coverage that aligned with the diversity of the communities. Next, I used the null model for each data set to ask how much sequencing effort was required to obtain higher levels of coverage. To obtain 95% and 99% coverage, an average of 3- and 101-fold more sequence data were estimated to be required than was required to obtain 90% coverage, respectively (Fig. 7). As suggested by the simulated coverages curve in Fig. 7, these estimates are conservative. Regardless, the sequencing effort required to achieve higher sequencing depth would likely limit the number of samples that could be sequenced when controlling for costs. Although it may be disconcerting to use rarefaction to normalize to a sequencing depth that is considerably lower than that obtained for the best sequenced community in a data set, sequencing coverage for many environments is probably adequate even at the lower sequencing depth. Of course, regardless of the concerns surrounding the choice of the rarefaction depth, the results throughout this study demonstrate that rarefaction is necessary to avoid reaching incorrect inferences.

## DISCUSSION

Over the past decade, the question of whether to use rarefaction with microbial community sequence data has become controversial. The analyses I presented here strongly indicate that rarefaction is necessary to control for uneven sequencing effort when comparing communities based on alpha and beta diversity indices. Compared to all other methods, rarefaction was the most consistent method for removing the correlation between sequencing depth and alpha or beta diversity metrics even when the sequencing depth varied by as much as 97-fold across samples. I showed that this correlation could lead falsely detecting differences between treatment groups if sequencing depth and sequencing effort are confounded. The correlation with sequencing effort leads to an artificial increase in the variation between samples and a reduced power to detect true differences in alpha and beta diversity. For these reasons, rarefaction is a valuable tool to control for uneven sequencing effort until improved statistical procedures are developed or it becomes possible to more evenly distribute sequencing effort across samples.

Historically, the primary alternative to rarefaction for measuring alpha diversity is to estimate the metric using non-parametric or parametric models with raw counts and to then compare the estimates (7, 14). My results demonstrate that such approaches are limited for several reasons. First, non-parametric richness estimates such as ACE and Chao1 are sensitive to sequencing effort. Therefore, these estimators do not, in practice, remove the effects of sequencing effort. Second, parametric approaches, such as those implemented in the breakaway R package, generate confidence intervals that are likely to include the true richness and theoretically shrink with increased sequencing effort. Yet for most samples, the confidence intervals around the estimates are too wide to be meaningful, again leading to an inability to remove the effects of sequencing effort. Third, it has become an increasingly common practice for researchers to remove sequences that are rare in a sample (e.g., those that appear once). Although that approach was not taken in this study, removing rare sequences would skew the distribution of sequences and OTUs leading to a distortion of the measurement of alpha diversity (38). The effects of removing rare sequences would vary across samples depending on the number of sequences in each sample. One interesting result of this analysis was the demonstration that as metrics that depend less on rare taxa are used, the effect of uneven sequencing effort was reduced. For example, richness counts a sequence appearing once as much as sequence appearing 1,000 times, while the Shannon diversity index places more emphasis on the more abundant sequence, and the inverse Simpson index even more. Although normalizing communities to a common number of sequences is also suggested (e.g., SRS normalization) to control for uneven sequencing effort, the current analysis indicates that its performance does not meet that of rarefaction. Because alpha diversity metrics are so sensitive to sampling effort and residual PCR and sequencing artifacts, it is important to treat their values on a relative (e.g., 5% difference) rather than an absolute basis (e.g., difference of 48 OTUs). Rarefaction is an effective and consistent approach to control for uneven sequencing effort.

Use of relative abundances, normalized counts, variance stabilizing transformations, and centered log ratios have each been recommended as more robust alternatives to rarefaction. Again, the only approach that consistently removed the effects of uneven sequencing effort across alpha and beta diversity analyses was rarefaction. Many of the alternative methods have been borrowed from differential gene expression analysis. Unfortunately, there is an important but under appreciated difference between gene expression and community data. This is the interpretation of unobserved data. For gene expression analysis in a single organism, the lack of any sequencing data for a gene would indicate that although the gene was present, its expression was below the limit of detection. Sequencing the same organism under multiple conditions would not lead to a seemingly unbounded number of genes in the organisms. Rather, the number of genes has a definite limit that is knowable from the genome sequence. With microbiome data, an unobserved sequence could mean that the organism was present, but below the limit of detection or that the organism was missing. Because we have yet to exhaustively sample any community in the same way we have sequenced a single genome, it is unreliable to impute the presence of all organisms. Yet, this is exactly what the variance stabilization transformation and most CLR techniques do. This analysis has demonstrated a clear correlation between distances calculated by these methods and sequencing effort. This result is at odds with the claims by others that the distances are scale invariant (23, 25). Again, rarefaction is the most effective and consistent approach to control for uneven sequencing effort when calculating beta diversity metrics.

Two common critiques of rarefaction are that the approach omits valid data and that the selection of rarefaction threshold is arbitrary (11). I disagree with both critiques. All of the data are used to calculate the mean value of the metrics after repeatedly subsampling the data. When the data set is subsampled, every sequence has a random chance of being included in the calculated metric. When that subsampling is repeated a large number of times (e.g., 100 or 1,000) the risks of ignoring or oversampling rare taxa are mitigated. As for the second criticism, I would resist the claim that the selection of the rarefaction threshold is arbitrary. In practice, there is a tradeoff between sampling breadth and sequencing depth. Greater breadth will increase the statistical power to compare treatment groups and greater depth will increase the resolution to describe the communities. My personal process for selecting a rarefaction threshold involves looking for a natural break in the distribution of the number of sequences. For example, the Lake data set used in this study had a clear break around 10,000 sequences (Fig. S1). I would also consider what samples are below any threshold that I select. If there were critical samples below the threshold, I would either reduce the threshold or obtain more sequences from those samples. As shown in my analysis of Good’s coverage values, most studies obtain an ample level of coverage and would need to increase their sequencing depth by 10-fold to increase the coverage by several percentage points. In past work, I have favored increasing sampling breadth over sequencing depth.

An additional critique of rarefaction is that the variation between alpha diversity metrics shrinks as the rarefaction threshold decreases, which may compromise the power to detect differences between treatment groups. The iNEXT methods attempt to minimize this effect by estimating alpha diversity metrics for samples whose sequencing depth is below a threshold and using rarefaction on samples with a sequencing depth above that threshold (17). This threshold is set at either twice the size of the smallest sample (i.e., size-based) or by controlling for the coverage when the number of sequences in each sample is doubled. An example of this can be seen in Fig. S2 where richness values obtained by estimation are shown in gray and those by rarefaction are in black. While my data suggest that the size-based approach has promise for estimating alpha diversity, I found that it was not as consistent as rarefaction for smaller data sets where there was a narrow distribution in sequencing depths. This is likely because in these situations, the algorithm relies more on estimation than rarefaction.

The up to 100-fold difference in sample sizes is an unfortunate byproduct of how sequencing libraries are constructed. Researchers perform separate PCRs for each sample with unique index (aka, barcode) sequences that allow them to later assign sequences back to the samples that they came from. When the PCR products are pooled, efforts are often taken to pool the fragments in equimolar ratios using one of two approaches. First, they often will quantify the concentration of DNA from each PCR and then pool DNA in the desired amounts. Alternatively, they may use normalization plates where each well can hybridize a uniform amount of DNA that is then eluted and pooled. Clearly, both approaches have limitations that reduce the ability to truly achieve equimolar mixture. Although normalization procedures are expected to remove the relationship between sequencing depth and sample biomass (40), several recent studies have found a positive correlation (41, 42). Even in cases where there is no correlation, for some samples, it is common to co-amplify non-specific DNAs which are later removed during sequence processing (43). It is clear that better strategies are needed to reduce the variation in the number of sequences generated for each sample. Furthermore, it is important to keep in mind that because of residual sequencing errors, PCR artifacts, and incomplete sequencing, it is not possible to obtain absolute quantification of alpha or beta diversity values. Instead, comparisons of these values need to be made on a relative basis.

All simulations have weaknesses and should be interpreted with caution. Previous studies have reached different conclusions than I have drawn here and elsewhere (12). I believe that the differences are largely due to confusion over the differences in definitions of rarefaction, the types of analysis being used to test the effects of rarefaction, and the use of simulated and actual communities that represent extremes, which are rarely found in real studies. It is worth noting that others have also observed sequencing depth correlations with CLR-based distances, but indicated that the correlation was less than what was observed without rarefaction (e.g., see Fig. S3 from reference 23). The simulated communities generated and analyzed in this study had the advantage of being designed with known properties including the alpha and beta diversity and their differences between treatment groups. Furthermore, I have used a wide array of alpha and beta diversity metrics and tests of significance that are commonly used in microbial ecology studies. The result is the conclusion that it is perfectly admissible and proper to use rarefaction with microbial community data. The alternative is to risk reaching unwarranted conclusions.

## MATERIALS AND METHODS

### Choice of data sets

The specific studies used in this study were selected because their data were publicly accessible through the Sequence Read Archive, the original investigators sequenced the V4 region of the 16S rRNA gene using paired 250 nt reads, and my previous familiarity with the data. The use of paired 250 nt reads to sequence the V4 region resulted in a near-complete twofold overlap of the V4 region resulting in high quality contigs with a low sequencing error rate (3). These data were processed through a standardized sequence curation pipeline to generate OTUs using the mothur software package (3, 9). OTUs were identified using the OptiClust algorithm to cluster sequences together that were not more than 3% different from each other (44).

### Null community model

Null community models were generated such that within a data set, the number of sequences per sample and the number of sequences per OTU across all samples within the data set were the same as was observed in original. This model effectively generated statistical samples of a single community so that there should not have been a statistical difference between the samples. This model was implemented by randomly assigning each sequence in the data set to an OTU and sample while keeping constant the number of sequences per sample and the total “number of sequences in each OTU. This is a similar approach to that of the “IS” algorithm described by Ulrich and Gotelli (45). Because the construction of the null models was a stochastic process, 100 replicates were generated for each data set.

### Null treatment models

I created unbiased and biased treatment models. In the unbiased model, samples were randomly assigned to one of two treatment groups. I created two biased treatment models. In the first, samples that had more than the median number of sequences for a data set were assigned to one treatment group and the rest were assigned to a second treatment group (see Fig. S4). In the second, samples whose sequencing depth where in the fifth percentile or lower were assigned to one treatment group while those in the 95% percentile or higher were assigned to another; the remaining samples were randomly assigned to either treatment group (see Fig. 3). Regardless of how the null treatment models were constructed, comparison of any diversity metric across the two treatment groups should have only yielded a significant result in 5% of the simulations when testing under a type I error (i.e., α) of 0.05.

### Skewed abundance community model

In the skewed abundance community model, communities were randomly assigned to one of two simulated treatment groups. Communities in the first treatment group were generated by calculating the relative abundance of each OTU across all samples and using those values as the probability of sampling each OTU. This probability distribution was sampled, with replacement, until each sample had the same number of sequences that it did in the observed data. Samples in the second treatment group were generated by adjusting the relative abundances of the OTUs in the first treatment group by increasing the relative abundance of 10% of the OTUs by 5%. These values were determined after empirically searching for conditions that resulted in a large fraction of the randomizations yielding a significant result across most of the studies. Sequences were sampled from the skewed community community until each sample had the same number of sequences that it did in the observed data. Under the skewed abundance community model, each sample represented a statistical sampling of two communities such that there should not have been a statistically significant difference within a treatment group, but there was between the treatment groups. Because the construction of the skewed abundance community model was a stochastic process, 100 replicates were generated for each data set.

### Richness-adjusted community model

In the richness-adjusted community model, communities were randomly assigned to one of two simulated treatment groups. Communities in the first treatment group were generated by calculating the relative abundance of each OTU across all samples and using those values as the probability of sampling each OTU. This probability distribution was sampled until each sample had the same number of sequences that it did in the observed data. Samples in the second treatment group were generated by removing 3% of the OTUs from the data set and recalculating the relative abundance of the remaining OTUs. Sequences were sampled from the richness-adjusted community distribution, with replacement, until each sample had the same number of sequences that it did in the observed data. Under the richness-adjusted community model, each sample represented a statistical sampling of two communities such that there should not have been a statistically significant difference within a treatment group, but there was between the treatment groups. Because the construction of the richness-adjusted community model was a stochastic process, 100 replicates were generated for each data set.

### Test of statistical significance

Statistical comparisons of alpha diversity metrics across the simulated treatment groups were performed using the non-parametric two-sample Wilcoxon test as implemented in wilcoxon.test in the stats base R package. This test was selected because the alpha diversity metrics tended to not be normally distributed and each data set required a different transformation to normalize the data. Comparisons of beta diversity metrics were performed using the adonis2 function from the vegan (v.2.6.2) R package (10). The adonis2 function implements a non-parametric multivariate analysis of variance using distance matrices (46). Throughout this study, I used 0.05 as the threshold for assessing the statistical significance of any *P*-values.

### Power analysis

The parameters used to design the skewed abundance and richness-adjusted community models were set to impose a known effect size when using community data normalized by rarefaction. The statistical power to detect these differences was determined by calculating the *P*-value for each of 100 replicate simulated set of samples from each data set using the various alpha and beta diversity metrics. The percentage of tests that yielded a significant *P*-value was considered the statistical power (i.e., one minus the type II error) to detect the difference.

### Alpha diversity calculations

Various strategies for handling uneven sequencing effort were evaluated to identify the best approach for calculating community richness and Shannon and inverse Simpson diversity indices. OTU counts were used as input to calculate sample richness and Shannon and inverse Simpson diversity using mothur (9, 47). Shannon diversity was calculated as


$$H_{shannon}=-\sum_{i=1}^{S_{obs}}\frac{n_{i}}{N}ln\frac{n_{i}}{N}$$

The Simpson diversity was calculated as


$$D_{simpson}=\frac{\sum_{i=1}^{S_{obs}}n_{i}\left(n_{i}-1\right)}{N\left(N-1\right)}$$

The inverse Simpson diversity was calculated as $1/D_{simpson}$. In both formulae, $n_{i}$ was the number of sequences in OTU i and N was the number of sequences in the sample. Rarefaction of richness, Shannon diversity, and inverse Simpson diversity values were carried out in mothur. Briefly, mothur calculates each value on a random draw of the same number of sequences from each sample and obtains a mean value based on 1,000 random draws. SRS was used to normalize OTU counts to the size of the smallest sample in each data set using the SRS R package (v.0.2.3) (20). Normalized OTU counts were used to calculate sample richness and Shannon and inverse Simpson diversity values using mothur. Data normalized by CSS were not reported for alpha diversity values since the relative abundances of the features do not change with the normalization procedure (21). The non-parametric bias-corrected Chao1 and ACE richness estimators (16) and a non-parametric estimator of the Shannon diversity (15) were calculated using raw OTU counts with mothur. The iNEXT R package was used to estimate richness and Shannon and inverse Simpson diversity metrics with default parameters in the estimateD function (v.3.0.0) (17). In the size-based approach, estimateD estimates alpha diversity at a level that is twice the size of the smallest sample, and in the coverage-based approach, it does so at a level that matches the smallest predicted coverage when the number of sequences in each sample is doubled. Parametric estimates of sample richness were calculated using the breakaway R package (v.4.7.9) (18). My analysis reports both the results from running default model selection procedure and the Poisson model. The default model selection returned either the Kemp, negative binomial, or Poisson model. Relative abundance data were not used to calculate alpha diversity metrics since the richness and evenness do not change from the raw data when dividing each sample by the total number of sequences in the sample.

### Beta diversity calculations

Similar to the alpha diversity calculations, multiple approaches were used to control for uneven sequencing effort and calculate beta diversity. Raw and OTU counts were used for input to calculate the Jaccard, Bray-Curtis, and Euclidean dissimilarity indices using the vegdist function from the vegan R package (v.2.6.2) (10). The Jaccard index was calculated as


$$D_{Jaccard}=1-\frac{S_{AB}}{S_{A}+S_{B}-S_{AB}}$$

where $S_{A}$ and $S_{B}$ were the number of OTUs in samples A and B and $S_{AB}$ was the number of OTUs shared between the two samples. The Bray-Curtis index was calculated as


$$D_{Bray-Curtis}=1-\frac{\sum_{i=1}^{S_{T}}\left|n_{A,i}-n_{B,i}\right|}{N_{A}+N_{B}}$$

where $n_{A,i}$ and $n_{B,i}$ were the number of sequences observed in OTU i from samples A and B, respectively. $N_{A}$ and $N_{B}$ were the total number of sequences in samples A and B, respectively. $S_{T}$ was the total number of OTUs observed between the two samples. The Euclidean distance was calculated as


$$D_{Euclidean}=1-\sqrt{\sum_{i=1}^{S_{T}}{\left(n_{A,i}-n_{B,i}\right)}^{2}}$$

These metrics were calculated using the relative abundance of each OTU using the vegdist function from the vegan R package. The relative abundance was calculated as the number of sequences in the OTU (e.g., $n_{A,i}$) divided by the total number of sequences in the sample (e.g., $N_{A}$).

Beta diversity values generated with rarefaction were calculated using the avgdist function in vegan. Briefly, vegan’s avgdist function calculates each pairwise dissimilarity index after obtaining a random draw of the same number of sequences from each sample. After obtaining 100 random draws, it returns the mean value.

Three approaches were taken to normalize the number of sequences across samples within a data set. SRS and CSS were used to normalize raw OTU counts using the SRS (v.0.2.3) and metagenomeSeq (v.1.36.0) R packages, respectively (20, 21). The normalized counts were then used to calculate Jaccard and Bray-Curtis dissimilarity indices. Finally, the VST, as implemented in the DESeq2 (v.1.34.0) R package, was used to normalize the data as described by McMurdie and Holmes (11, 48). Because the VST approach generated negative values, which are incompatible with calculating Jaccard and Bray-Curtis dissimilarity values, Euclidean distances were calculated instead.

Raw OTU counts were used to calculate CLR values for each OTU, which were then used to calculate Euclidean distances; such distances are commonly referred to as Aitchison distances. CLR abundances were calculated as


$$CLR\left(n_{j}\right)=\left[ln\frac{x_{ij}}{g\left(x_{j}\right)},...,ln\frac{x_{S_{T}j}}{g\left(x_{j}\right)}\right]$$

where $x_{ij}$ was the number of sequences observed for OTU i in sample j and $g\left(\right)$ was the geometric mean; $x_{j}$ was the count of the $S_{T}$ OTUs in sample j. Because the geometric mean is zero if any OTU is absent from a sample, the CLR is undefined when there are unobserved OTUs in a sample. To overcome this problem, I attempted four approaches. The first, One CLR, added a pseudo-count of 1 to the abundance of all OTUs (19, 21). The second, Nudge CLR, added a pseudo-count of 1 divided by the total number of sequences in a sample to each OTU in the sample (19, 24). The third, Zero CLR, imputed the value of the zeroes based on the observed data using the zCompositions (v.1.4.0.1) R package (49). The final approach, Robust PCA, calculated the CLR on non-zero counts and then used matrix completion to replace the zero counts; this was implemented using the rpca function in the Gemelli Python package (v.0.0.9) with default parameters (23). It was not possible to perform Robust PCA on the Rice, Seagrass, or Stream data sets since more than 250 GB of RAM was required; the other data sets required less than 16 GB of RAM.

### Analysis of sequencing coverage

To assess the level of sequencing coverage, I calculated Good’s coverage ($C_{Good}$) using mothur:


$$C_{Good}=100\%\times \left(1-\frac{n_{1}}{N_{T}}\right)$$

where $n_{1}$ was the number of OTUs with only one sequence in the sample and $N_{T}$ was the total number of sequences in the sample. Good’s coverage was calculated (i) using the observed OTU counts for each sample and data set, (ii) following rarefaction (1,000 iterations) of the observed OTU counts to the size of the smallest sample in each data set, and (iii) after rarefaction (1,000 iterations) of the null community distribution.

### Reproducible data analysis

A complete reproducible workflow written in Snakemake (v.7.15.2) and Conda (v.4.12.0) computational environment can be obtained from the GitHub hosted git repository for this project (https://github.com/SchlossLab/Schloss_Rarefaction_mSphere_2024). This paper was written in R-flavored markdown (v.2.16) with the kableExtra (v.1.3.4) package. The mothur (v.1.47.0) and R (4.1.3) software packages were used for all analyses with extensive use of functions in the tidyverse metapackage (v.1.3.1). A preliminary version of this analysis was presented as the Rarefaction video series on the Riffomonas Project YouTube channel (https://www.youtube.com/playlist?list=PLmNrK_nkqBpJuhS93PYC-Xr5oqur7IIWf).

## SUPPLEMENTAL MATERIAL

Supplemental figures msphere.00354-23-s0001.pdf Figures S1 to S4.

ASM does not own the copyrights to Supplemental Material that may be linked to, or accessed through, an article. The authors have granted ASM a non-exclusive, world-wide license to publish the Supplemental Material files. Please contact the corresponding author directly for reuse.
