# Supplementary material for: Rarefaction is currently the best approach to control for uneven sequencing effort in amplicon sequence analyses
Source: mSphere. 2024 Jan 22;9(2):e00354-23. doi: 10.1128/msphere.00354-23 (PMC10900887; doi:10.1128/msphere.00354-23)
Supplement: Supplemental figures — Figures S1 to S4. [file msphere.00354-23-s0001.pdf]

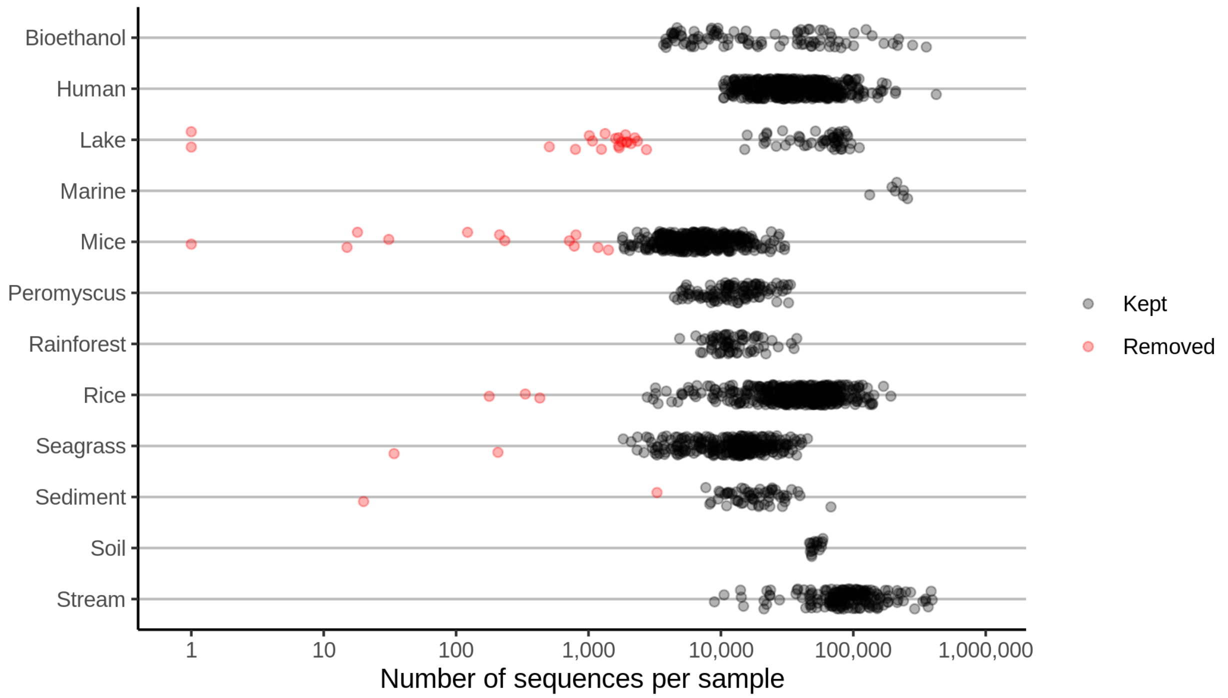

Figure S1. The number of sequences observed in each sample for each dataset included in this analysis generally varied by 10 to 100-fold. The threshold for specifying the number of sequences per sample varied by dataset and was determined based on identifying natural breaks in the data. This figure is similar to Figure S1 of (28)

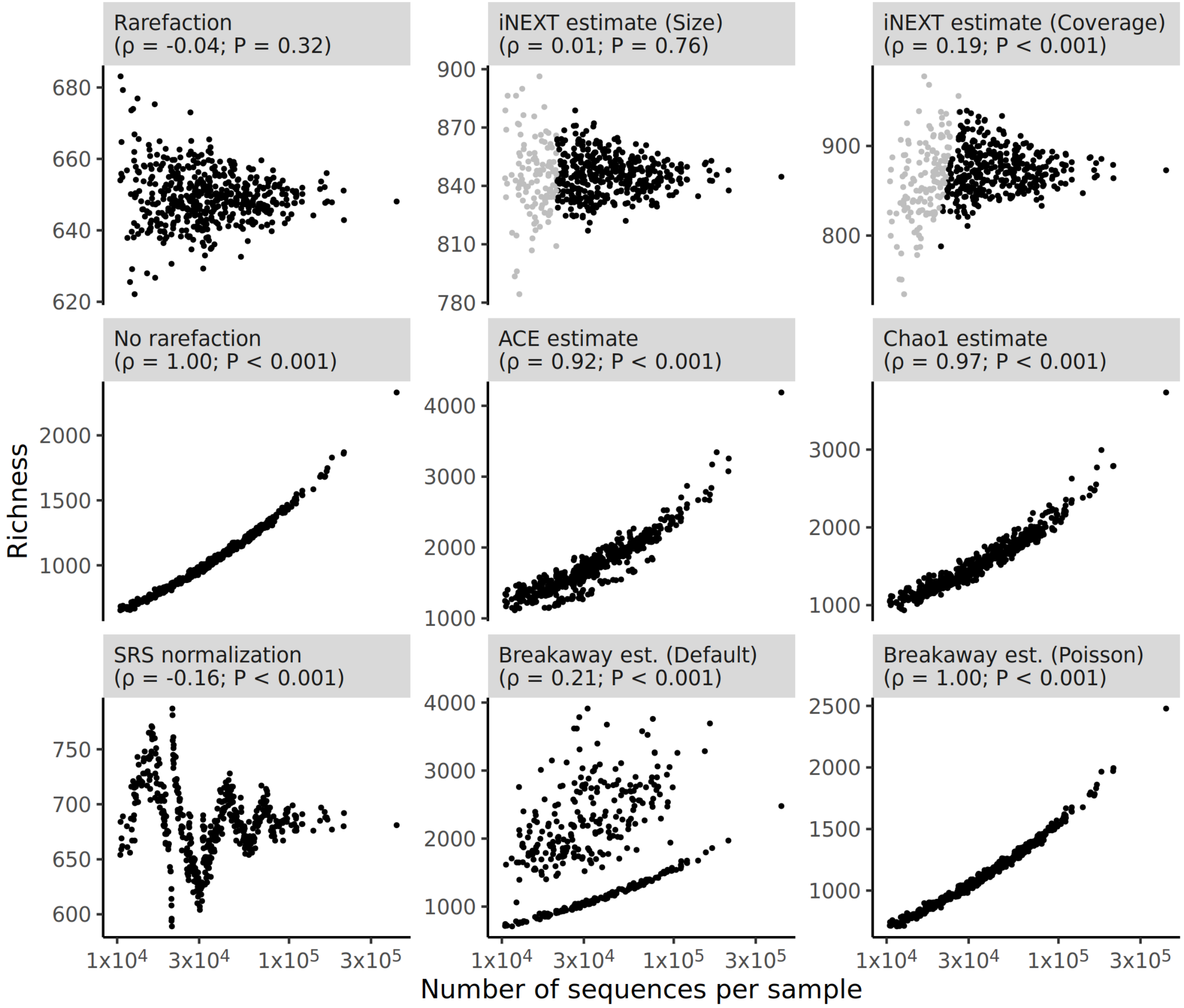

Figure S2. Examples of the richness in each of the 490 samples that were generated for one randomization of the null model using the human dataset. The x-axis indicates the number of sequences in each of the samples prior to each method's approach of controlling for uneven sequencing effort. The Spearman correlation coefficient (ρ) and test of whether the coefficient was significantly different from zero are indicated for each panel. The gray points for the iNEXT estimates indicate samples where richness values were estimated while the black points are those were rarefaction was used.

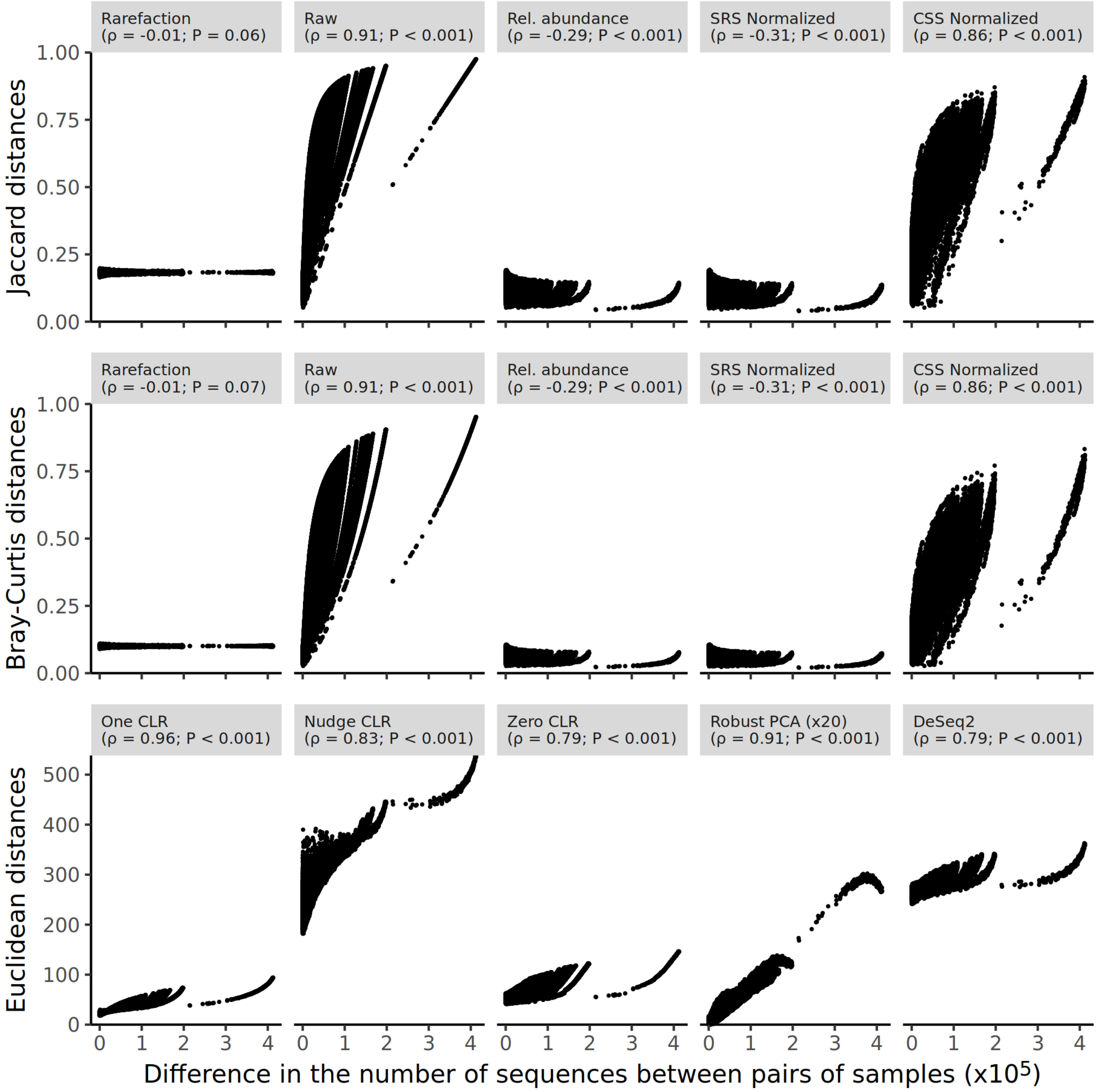

Figure S3. Examples of differences in beta diversity in each of the 490 samples that were generated for one randomization of the null model using the human dataset. The x-axis indicates the difference in the number of sequences in each of the samples that went into calculating the pairwise distance prior to each method's approach of controlling for uneven sequencing effort. The values on the y-axis for the Robust PCA distances were multiplied by 20 to fit them on the same scale as the other methods. The Spearman correlation coefficient (ρ) and test of whether the coefficient was significantly different from zero are indicated for each panel.

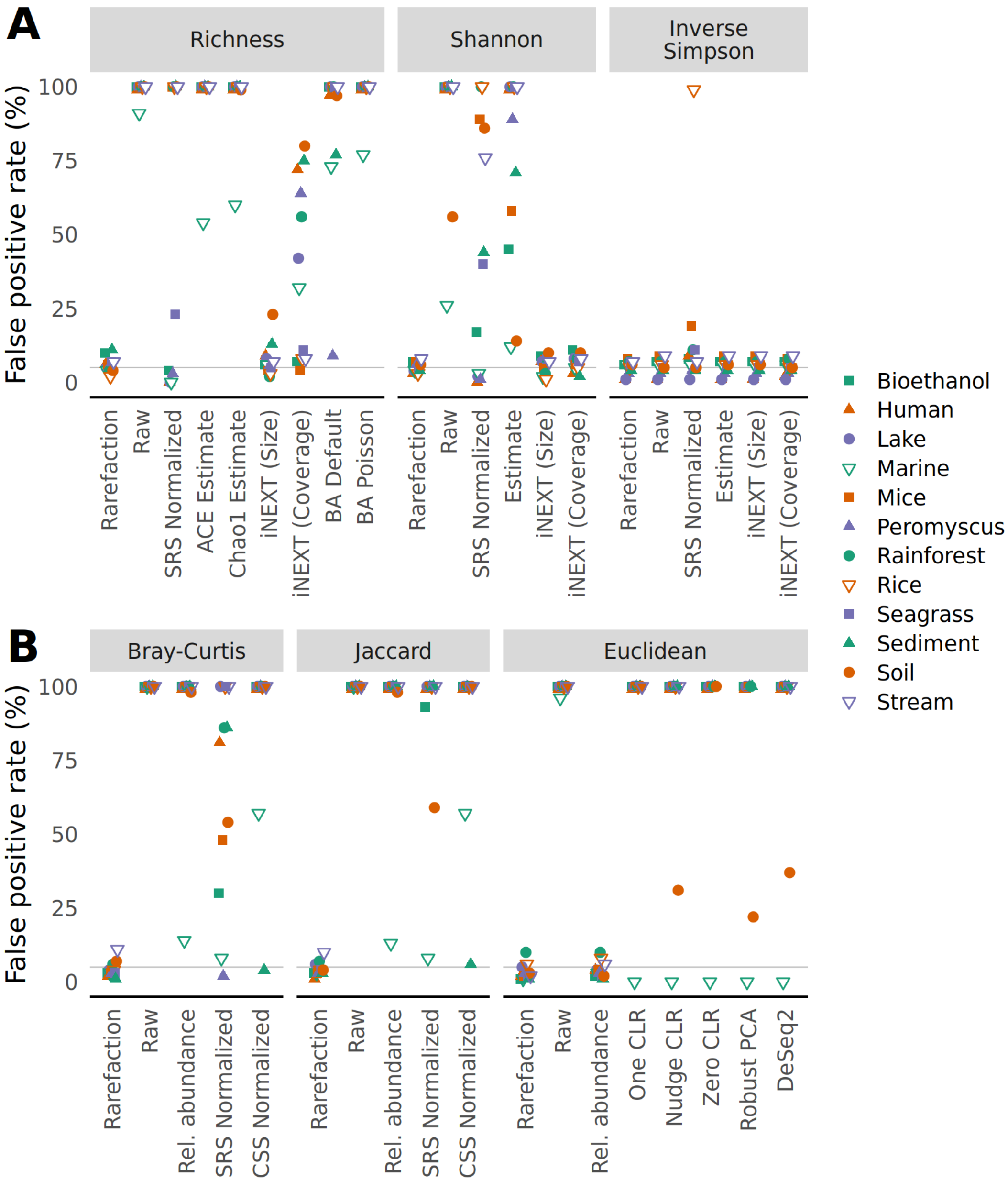

Figure S4.The risk of falsely detecting a difference between treatment groups drawn from a null model did not meaningfully vary from 5% when data are normalized by rarefaction when sequencing depth was completely confounded with treatment group. Samples were assigned to different treatment groups based on whether they were above the median number of sequences for each dataset. To calculate the false detection rate, datasets were regenerated 100 times and differences in alpha diversity were tested using a Wilcoxon test (A) and differences in beta diversity were tested using PERMANOVA (B) at a 5% threshold. The false positive rate was the number of times a dataset yielded a significant result.
